# The Effects of Sleep Quality and Resilience on Perceived Stress, Dietary Behaviors, and Alcohol Misuse: A Mediation-Moderation Analysis of Higher Education Students from Asia, Europe, and North America during the COVID-19 Pandemic

**DOI:** 10.3390/nu13020442

**Published:** 2021-01-29

**Authors:** Chen Du, Megan Chong Hueh Zan, Min Jung Cho, Jenifer I. Fenton, Pao Ying Hsiao, Richard Hsiao, Laura Keaver, Chang-Chi Lai, HeeSoon Lee, Mary-Jon Ludy, Wan Shen, Winnie Chee Siew Swee, Jyothi Thrivikraman, Kuo-Wei Tseng, Wei-Chin Tseng, Stephen Doak, Sara Yi Ling Folk, Robin M. Tucker

**Affiliations:** 1 Department of Food Science and Human Nutrition, Michigan State University, East Lansing, MI 48824, USA; duchen@msu.edu (C.D.); imigjeni@msu.edu (J.I.F.); folksara@msu.edu (S.Y.L.F.); 2 Division of Nutrition and Dietetics, International Medical University, Kuala Lumpur 57000, Malaysia; megan_chong@imu.edu.my (M.C.H.Z.); winnie_chee@imu.edu.my (W.C.S.S.); 3 Global Public Health, Leiden University College, 2595 DG The Hague, The Netherlands; m.j.cho@luc.leidenuniv.nl (M.J.C.); j.k.thrivikraman@luc.leidenuniv.nl (J.T.); 4 Department of Food and Nutrition, Indiana University of Pennsylvania, Indiana, PA 15705, USA; pyhsiao@iup.edu; 5 Department of Kinesiology, Health, and Sport Science, Indiana University of Pennsylvania, Indiana, PA 15705, USA; hsiao@iup.edu; 6 Department of Health and Nutritional Science, Institute of Technology Sligo, F91 YW50 Sligo, Ireland; keaver.laura@itsligo.ie (L.K.); stephen.doak@mail.itsligo.ie (S.D.); 7 Department of Exercise and Health Sciences, University of Taipei, Taipei 11153, Taiwan; sports_injury0406@yahoo.com.tw (C.-C.L.); fossil0405@yahoo.com.tw (K.-W.T.); speedceng@gmail.com (W.-C.T.); 8 Department of Human Services, Bowling Green State University, Bowling Green, OH 43403, USA; leeh@bgsu.edu; 9 Department of Public and Allied Health, Bowling Green State University, Bowling Green, OH 43403, USA; mludy@bgsu.edu (M.-J.L.); wanshen@bgsu.edu (W.S.)

**Keywords:** food intake, nutrition, substance use, college, university students, COVID-19, pandemic, health behaviors, mental health

## Abstract

Background: The coronavirus disease 2019 (COVID-19) pandemic has increased the already high levels of stress that higher education students experience. Stress influences health behaviors, including those related to dietary behaviors, alcohol, and sleep; yet the effects of stress can be mitigated by resilience. To date, past research studying the connections between dietary behaviors, alcohol misuse, sleep, and resilience commonly investigated singular relationships between two of the constructs. The aim of the current study was to explore the relationships between these constructs in a more holistic manner using mediation and moderation analyses. Methods: Higher education students from China, Ireland, Malaysia, South Korea, Taiwan, the Netherlands, and the United States were enrolled in a cross-sectional study from April to May 2020, which was during the beginning of the COVID-19 pandemic for most participants. An online survey, using validated tools, was distributed to assess perceived stress, dietary behaviors, alcohol misuse, sleep quality and duration, and resilience. Results: 2254 students completed the study. Results indicated that sleep quality mediated the relationship between perceived stress and dietary behaviors as well as the relationship between perceived stress and alcohol misuse. Further, increased resilience reduced the strength of the relationship between perceived stress and dietary behaviors but not alcohol misuse. Conclusion: Based on these results, higher education students are likely to benefit from sleep education and resilience training, especially during stressful events.

## 1. Introduction

Globally, higher education students report high levels of stress [1,2,3], which increases the risk of engaging in unhealthy behaviors [4,5]. Given these high levels of stress, reports from recent studies suggesting the coronavirus disease 2019 (COVID-19) pandemic worsened stress among students in higher education in multiple countries is especially concerning [6,7,8,9]. Unhealthy behaviors related to stress include both poor dietary behaviors [10,11,12,13] and alcohol misuse [14], which can lead to health and social risks [15]. Sleep and psychological resilience are two factors that have the potential to alter the relationships between stress and health behaviors. Stress negatively influences sleep [16,17,18] while short sleep and poor sleep quality increase undesirable dietary behaviors, such as increased energy intake and more frequent consumption of sweets [19,20,21]. Additionally, short sleep and poor sleep quality increase the risk of alcohol misuse [22]. Thus, sleep may serve as a mediator in these relationships. Besides sleep, psychological resilience has also been shown to affect the relationships between stress and health behaviors [23,24]. Psychological resilience, defined as the processes of adapting and coping quickly when facing a significant source of stress [25], reduces the negative effect of stress on health behaviors by improving the ability to cope and manage stress [23,24,26,27]. Based on previous work, it is clear that stress, dietary behaviors, alcohol misuse, sleep, and resilience are interconnected.

Current research studying the relationships between these variables commonly investigates a singular relationship between two of the constructs [14,16,17,19,20,23,24,28]; however, the relationships between these constructs are far more complicated. Thus, a more comprehensive understanding of the relationships between stress, dietary behaviors, alcohol misuse, sleep, and resilience is needed to better guide the development of programming to improve the health of higher education students.

To improve our understanding of the interactions between these variables, mediation and moderation analyses were conducted. Mediation analysis examines whether the relationship between an independent variable and a dependent variable is related to an intermediate variable (mediator) [29], and moderation analysis determines whether the direction and strength of the relationship between two variables are subject to a moderating variable [30]. The objectives of the study were to examine whether (1) sleep quality or duration mediated the relationship between perceived stress and dietary behaviors, (2) sleep quality or duration mediated the relationship between perceived stress and alcohol misuse, (3) resilience moderated the direct and the indirect relationships between perceived stress and dietary behaviors, (4) resilience moderated the direct and the indirect relationships between perceived stress and alcohol misuse. Based on the documented relationships between stress, dietary behaviors, alcohol misuse, sleep, and resilience, hypotheses included:

(1) Higher levels of perceived stress were likely to be associated with poorer dietary behaviors through decreased sleep quality and sleep duration.
(2) Higher levels of perceived stress were likely to be associated with higher alcohol misuse through decreased sleep quality and sleep duration.
(3) Higher levels of resilience were likely to reduce the negative effects of perceived stress on poor dietary behaviors.
(4) Higher levels of resilience were likely to reduce the negative effects of perceived stress on alcohol misuse.

## 2. Materials and Methods

### 2.1. Study Design

Undergraduate and graduate students who were at least 18 years old, studying at universities in China, Ireland, Malaysia, Taiwan, South Korea, the Netherlands, and the United States (U.S.), were recruited for this study. Students who were less than 18 years old and were not currently enrolled in the surveyed universities were excluded from the study. Students were recruited via university research advertisement systems. The study took place in April and May 2020 during which time period most states in the U.S. and most areas in Ireland, Malaysia, and the Netherlands had adopted “shelter in place” orders. Most areas of China and Taiwan were under “shelter in place” orders, but some areas had lifted the order. Cross-sectional data were collected online via survey platforms, and all surveys were administered in English.

The study was approved by the Michigan State University Human Research Protection Program (East Lansing, MI, USA), STUDY00004285, 4/7/2020; International Medical University Joint Committee on Research and Ethics (Kuala Lumpur, Malaysia), 481/2020, 5/14/2020; Faculty of Governance and Global Affairs Ethics Committee (The Hague, South Holland, Netherlands), 2020-009-LUC-Cho, 5/25/2020; Indiana University Institutional Review Board for the Protection of Human Subjects (Indiana, PA, USA), IRB Log 20-101, 5/15/2020; Institute Research Ethics Committee, Institute of Technology, Sligo (Sligo, Ireland), ref 2020015, 5/5/2020; University of Taipei Institutional Review Board (Taipei, Taiwan), IRB-2020-045, 6/16/2020; and Bowling Green State University Office of Research Compliance (Bowling Green, OH, USA;1599753 (US students), 4/29/2020; 1599753 (Chinese students), 5/22/2020; 1599753 (Korean students), 5/11/2020. Consent was obtained from all participants prior to the start of the study.

### 2.2. Demographics and Biological Information

Age, sex, class status (undergraduate vs. graduate), citizen status (international vs. domestic), and self-reported weight and height were collected. Graduate students included those pursuing masters, doctoral, and professional degrees. International students were attending a higher education institution in a country other than their citizenship; whereas, domestic students were attending a higher education institution in the country of their citizenship. Body mass index (BMI) was calculated from the reported weight and height.

### 2.3. Assessment of Perceived Stress and Resilience

Perceived stress was assessed using the Perceived Stress Scale-10 (PSS-10). The PSS-10 is a validated survey [31] that provides a score ranging from 0 to 40. Based on PSS-10 scoring, scores from 0 to 13, 14 to 26, and 27 to 40 indicate low, moderate, and high levels of stress, respectively.

Resilience was measured using the Brief Resilience Scale (BRS), which is a validated tool that measures psychological resilience [32]. The global resilience score ranges from 0 to 5, with a higher score indicating higher resilience.

### 2.4. Assessment of Dietary Behaviors and Alcohol Misuse

Dietary behaviors were assessed using an eight-item simplified food frequency questionnaire (FFQ)—Starting the Conversation (STC) [33]. The STC provides a global score of dietary behaviors ranging from 0 to 16; the higher the score, the more dietary behaviors that are not consistent with health.

Alcohol misuse was assessed using the Alcohol Use Disorders Identification Test Alcohol Consumption questionnaire (AUDIT-C), which provides a global alcohol misuse score. The AUDIT-C has been validated in many countries among university students [34,35,36]. A standard drink equivalents reference chart was provided at the beginning of the AUDIT-C survey section to help participants quantify drink servings [37] AUDIT-C scores range from 0 to 12, with a score of 4 and higher for men and 3 and higher for women indicating alcohol misuse [38]. Survey logic was set to show these questions only to students who met the minimum drinking age.

### 2.5. Assessment of Sleep Quality and Duration

Subjective sleep quality was assessed using the Pittsburgh Sleep Quality Index (PSQI), which has been well validated in many populations, including university students [39,40,41,42]. PSQI scores range from 0 to 21. Higher scores indicate poorer sleep quality, and a score of ≥5 indicates poor sleep quality [43]. Weekday and weekend sleep duration were assessed by asking participants how many hours they usually sleep during the weekdays and weekends. Average sleep duration was calculated using the equation, ((weekday sleep duration × 5) + (weekend sleep duration × 2))/7. Participants were classified as meeting sleep duration guidelines if they reported sleeping at least 7 h/night [44].

### 2.6. Assessment of the Influence of the COVID-19 Pandemic on the Factors Described Above

Questions about how the COVID-19 pandemic affected participants’ dietary intake, alcohol consumption, perceived stress, resilience, sleep quality, and sleep duration were asked. Examples of questions included: Are your answers to the perceived stress questions affected by the COVID-19 pandemic? Possible answers included: during the pandemic, I have less perceived stress than usual; during the pandemic, I have more perceived stress than usual; no change of stress during the pandemic. Has the COVID-19 pandemic influenced your alcohol intake? Possible answers included: yes, I have been drinking more; yes, I have been drinking less; no, it has not influenced my alcohol intake; I don’t drink alcohol.

### 2.7. Meditation and Moderation Models

**Figure 1:**
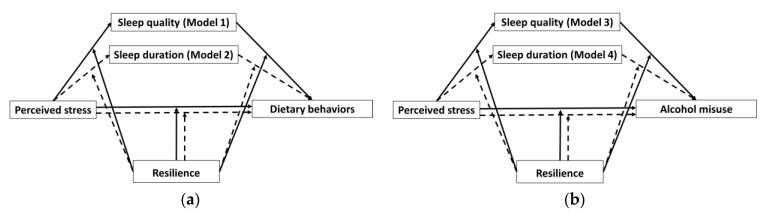
Proposed moderated mediation models. (**a**) Perceived stress on dietary behaviors through the mediation of sleep quality and the moderation of resilience (Model 1, solid line) and perceived stress on dietary behaviors through the mediation of sleep duration and the moderation of resilience (Model 2, dashed line). (**b**) Perceived stress on alcohol misuse through the mediation of sleep quality and the moderation of resilience (Model 3, solid line) and perceived stress on alcohol misuse through the mediation of sleep duration and the moderation of resilience (Model 4, dashed line).

To test the hypotheses stated in the introduction, four moderated mediation models were proposed (Figure 1).

### 2.8. Statistical Analysis

Only completed surveys were included in data analysis, and data were analyzed using IBM SPSS Version 26 (IBM Corporation, Armonk, New York, USA). Descriptive statistics were performed, and data were presented in percentage (%) or mean ± standard deviations (SD). Zero-order correlations were conducted to examine the relationships between all constructs included in mediation and moderation analysis models. Statistical significance was determined using Bonferroni adjusted *p* values. A total of 28 comparisons were performed in the correlation analysis; therefore, statistical significance was determined at *p* < 0.0018 (0.05 ÷ 28).

Moderated mediation analyses were conducted using the SPSS PROCESS Macro developed by Hayes [30]. All models were adjusted for age, sex, and BMI. The number of bootstraps performed for bias corrected bootstrap confidence intervals was set at 10,000. All variables entered in the models were approximately normally distributed after excluding outliers, which were defined by above or below mean ± 3SD. PROCESS was performed for each model by first selecting Model 59 [30,45] and then entering one independent variable (perceived stress), one mediator (sleep quality or sleep duration), one moderator (resilience), and one dependent variable (dietary behaviors or alcohol misuse). Statistical significance was determined by *p* < 0.05 for all analyses and the 95% confidence interval (CI) not crossing zero for the indirect effect testing of the mediation analyses.

To interpret the results of the mediation analyses, the following conditions were used. (1) A variable is considered a mediating variable when a significant indirect effect of an independent variable on a dependent variable through the mediator (bootstrap results) is noted. (2) The singular direct relationships between an independent, mediating, and dependent variable do not need to be significant in order for the mediation effect to be significant [30].

For moderation analyses, the following principles were applied when conducting the analyses. (1) For significant moderation effects, a statistically significant transition point can be identified using the Johnson-Neyman method [46]. (2) A moderator can moderate the relationship between an independent and a dependent variable directly or indirectly. Direct effects of a moderator are observed when the relationship between the independent and dependent variables is conditional to the moderator while holding the mediator constant. In contrast, testing for indirect effects of a moderator examines two conditional relationships. First, testing examines whether the relationship between an independent variable and a mediator is conditioned to a moderator. Second, testing examines whether the relationship between a mediator and a dependent variable is conditioned to a moderator. In summary, the differences between direct and indirect effects are that an indirect effect includes the mediator in the analysis while the direct effect does not.

To interpret the results of the moderation analyses, the following condition was used. Significant moderation between any two variables depends on the significant association between the two variables. For example, if a moderator significantly moderates the relationship between an independent variable and a mediator, then the relationship of the independent variable on the mediator must be significant [30].

## 3. Results

### 3.1. Demographics

A total of 2663 students initiated the survey, and a total of 2254 students completed the study and were included in the data analysis (Table 1). The completion rate for the study was 84.6%. The majority of the students were female (66.7%), undergraduate (79.9%), domestic (87.0%), and studying in the U.S. (58.7%) (Table 2). Social restrictions varied between the locations during the data collection period, but all locations surveyed adopted an online instructional format for at least some classes.

### 3.2. Correlations between Examined Variables

The Pearson zero-order correlation analyses showed that dietary behaviors were positively correlated with perceived stress and sleep quality, negatively correlated with resilience, and not correlated with sleep duration (Table 3). These findings indicate that poorer dietary behaviors (higher STC scores) were associated with greater perceived stress (higher PSS-10 scores), poorer sleep quality (higher PSQI scores), and lower resilience (lower BRS scores). Alcohol misuse was positively correlated with greater perceived stress and poorer sleep quality but not correlated with sleep duration and resilience. Additionally, dietary behavior scores and alcohol misuse scores were not correlated. Perceived stress was positively associated with sleep quality and negatively associated with resilience, which means that greater perceived stress was associated with poorer sleep quality and less resilience. Further, both sleep quality and sleep duration as well as sleep quality and resilience were negatively associated. These results indicate that poorer sleep quality was associated with shorter sleep duration and less resilience. For the covariates, age was negatively associated with perceived stress and sleep duration, but positively associated with resilience, which means that younger age was associated with greater perceived stress, longer sleep duration, and less resilience. Additionally, BMI was positively associated with dietary behaviors, alcohol misuse, perceived stress, sleep quality, and age, which indicates that higher BMI was associated with poorer dietary behaviors, more frequent alcohol consumption, greater perceived stress, poorer sleep quality, and older age.

### 3.3. Health Behavior Classification of Higher Education Students

More than three fourths of students were classified as experiencing moderate or high stress according to the PSS-10 [31] (Table 4). The majority of students were classified as poor sleepers according to the PSQI [43]; however, the majority of students also met the minimum recommended sleep duration of 7 h per night. In terms of alcohol misuse, over one-fourth of male students were classified as alcohol misusers while more than one-fifth of female students were classified as alcohol misusers according to the AUDIT-C [34].

### 3.4. Influence of the COVID-19 Pandemic on Health Behaviors and Mental Health of Higher Education Students

Participants’ self-assessments indicated that the COVID-19 pandemic had negative impacts on the diet and sleep quality of higher education students but not on alcohol consumption and sleep duration. More than one-third of the students reported worsened diet, and close to one-third of the students reported worsened sleep quality during the COVID-19 pandemic compared to before (Table 5). However, less than 20% of students reported increased alcohol consumption, and more than 20% of students reported drinking less during the COVID-19 pandemic compared to before. Additionally, close to half of students reported sleeping more during the COVID-19 pandemic compared to before. The net effect, which was the difference between students who reported worse/better and more/less, showed that more students reported diet and sleep quality to be worse than before, but alcohol use was lower, and sleep duration was longer compared to before the pandemic.

In terms of mental health, close to two-thirds of the students reported more perceived stress and close to one-third of the students reported less resilience during the COVID-19 pandemic compared to before. The net effect indicated that more students reported perceived stress and resilience to be worse than before.

### 3.5. Mediation and Moderation Analysis

The mediation analysis of Model 1 showed a significant direct effect of perceived stress on dietary behaviors, and perceived stress and dietary behaviors were positively correlated (*p* < 0.001). Perceived stress was also positively associated with sleep quality scores (*p* < 0.001), which means that as perceived stress increased, sleep quality decreased since higher PSQI scores indicate worse sleep quality (Table 6). Sleep quality was not associated with dietary behaviors in this model (*p* = 0.916). However, sleep quality significantly mediated the relationship between perceived stress and dietary behaviors, which was evidenced by the bootstrap result showing a significant indirect effect of perceived stress on dietary behaviors through its effect on sleep quality (B = 0.02, 95% CI 0.008 to 0.023). Based on the mediation conditions described in the methods section, sleep quality was still a significant mediator, even though sleep quality was not associated with dietary behaviors.

Two moderation pathways were included in the moderation analyses of whether resilience moderated the relationship between perceived stress and dietary behaviors directly or indirectly through sleep quality (Table 7). The number of paths reflects the number of outcome variables (sleep quality and dietary behaviors) [30]. Path 1 analysis indicated that resilience significantly moderated the relationship between perceived stress and sleep quality, based on the significant interaction effect of perceived stress and resilience on sleep quality (*p* < 0.001). As resilience increased, the positive association between perceived stress and sleep quality weakened per the Johnson-Neyman test of indirect effect for Path 1. Further, resilience indirectly moderated the relationship between perceived stress and dietary behaviors by moderating the relationship between perceived stress and sleep quality (*p* < 0.001). The results of the Path 2 analysis indicated that resilience directly moderated the relationship between perceived stress and dietary behaviors, as indicated by the significant interaction effect of perceived stress and resilience on dietary behaviors (*p* = 0.023). As resilience increased, the association between perceived stress and dietary behaviors weakened, and the association disappeared at the resilience score of 3.87 per the Johnson-Neyman test of direct effect for Path 2. Of note, 18.3% of students reported a resilience score above 3.87 on the 5-point scale, while 81.7% scored below based on the Johnson-Neyman test. However, resilience did not indirectly moderate this relationship by moderating the relationship between sleep quality and dietary behaviors because the interaction effect of sleep quality and resilience on dietary behaviors was not significant (*p* = 0.313).

To summarize, the mediation and moderation analysis of Model 1 showed that perceived stress and dietary behaviors were positively correlated, and this relationship was associated with sleep quality. This means that greater perceived stress was associated with poorer dietary behaviors, and the negative effect of perceived stress on dietary behaviors was related to poor sleep quality. Increased resilience may serve as a buffer to weaken the relationship between perceived stress and dietary behaviors, and the direct relationship between perceived stress and dietary behaviors diminished when resilience scores were above 3.87.

The mediation analysis of Model 2 showed a significant direct effect of perceived stress on dietary behaviors, and perceived stress and dietary behaviors were positively correlated (*p* < 0.001). Perceived stress was also negatively associated with sleep duration (*p* = 0.005) (Table 8). However, sleep duration was not associated with dietary behaviors in this model (*p* = 0.726). Additionally, sleep duration did not mediate the relationship between perceived stress and dietary behaviors as indicated by the non-significant bootstrap results (B = −0.002, 95% CI −0.004 to 0.0002) of testing whether perceived stress indirectly associated with dietary behaviors through its relationship with sleep duration.

Two moderation pathways were included in the moderation analyses of whether resilience moderated the relationship between perceived stress and dietary behaviors directly or indirectly through sleep duration (Table 9). Path 1 analysis indicated that resilience did not moderate the relationship between perceived stress and sleep duration since the interaction term of perceived stress and resilience was not associated with sleep duration (*p* = 0.09); therefore, the Johnson-Neyman test was not performed for Path 1. Path 2 analysis indicated that resilience directly moderated the relationship between perceived stress and dietary behaviors (*p* = 0.016), which was consistent with Model 1. Based on the Johnson-Neyman test of the direct effect for Path 2, as resilience increased, the association between perceived stress and dietary behaviors weakened, and the association disappeared at the resilience score of 4.16 (Table 9). In this instance, 10.3% of students reported a resilience score above 4.16 on the 5-pont scale based on the Johnson-Neyman test. The cut-off resilience score was slightly higher compared to Model 1 due to differences in constructs included in the models (sleep quality vs. sleep duration). However, resilience did not indirectly moderate the relationship between perceived stress and dietary behaviors by moderating the relationship between sleep duration and dietary behaviors because the interaction effect of sleep duration and resilience on dietary behaviors was not significant (*p* = 0.851).

To summarize, the mediation and moderation analysis of Model 2 demonstrated that perceived stress and dietary behaviors were positively correlated, and this was not associated with sleep duration. This means that greater perceived stress was associated with poorer dietary behaviors, and the negative effect of perceived stress on dietary behaviors was not related to short sleep duration. Once again, greater resilience weakened the relationship between perceived stress and dietary behaviors.

The mediation analysis of Model 3 showed that perceived stress positively correlated with sleep quality (*p* < 0.001), but perceived stress was not correlated with alcohol misuse (*p* = 0.299), and sleep quality was also not associated with alcohol misuse (*p* = 0.109) (Table 10). However, sleep quality significantly mediated the relationship between perceived stress and alcohol misuse, given the significant mediation bootstrap result (B = 0.02, 95% CI 0.009 to 0.025). Based on the mediation conditions described in the methods section, the mediation effect was still significant, even though the association between sleep quality and alcohol misuse and the association between perceived stress and alcohol misuse were not significant.

Two moderation pathways were included in the moderation analyses of whether resilience moderated the relationship between perceived stress and alcohol misuse directly or indirectly through sleep quality (Table 11). Path 1 was the same as Path 1 in Model 1 and had the same results, where resilience moderated the relationship between perceived stress and sleep quality (*p* < 0.001). Path 2 analysis indicated that resilience did not moderate the relationship between perceived stress and alcohol misuse directly or indirectly because both the interaction effect of perceived stress and resilience on alcohol misuse (*p* = 0.112) and the interaction effect of sleep quality and resilience on alcohol misuse (*p* = 0.573) were not significant.

To summarize, the mediation and moderation analyses of Model 3 showed that perceived stress was associated with alcohol misuse only through its relationship with sleep quality. This means that greater perceived stress was associated with more frequent alcohol misuse, and the negative effect of perceived stress on alcohol misuse was related to poor sleep quality. Resilience did not serve as a buffer to alter the relationship between perceived stress and alcohol misuse, but it did weaken the relationship between perceived stress and sleep quality (PSQI).

The mediation analysis of Model 4 indicated that perceived stress was not correlated with sleep duration (*p* = 0.310) and alcohol misuse (*p* = 0.975), and sleep duration was not correlated with alcohol misuse (*p* = 0.789) (Table 12). Additionally, sleep duration did not mediate the relationship between perceived stress and alcohol misuse, which was evidenced by the non-significant mediation bootstrap result (B = 0.0002, 95% CI −0.0006 to 0.0013).

Two moderation pathways were included in the moderation analyses of whether resilience moderated the relationship between perceived stress and alcohol misuse directly or indirectly through sleep duration (Table 13). Path 1 was the same as Path 1 in Model 2. Path 1 analysis indicated that resilience did not moderate the relationship between perceived stress and sleep duration since the interaction term of perceived stress and resilience was not associated with sleep duration (*p* = 0.431); therefore, the Johnson-Neyman test was not performed for Path 1. The Path 2 moderation analyses of Model 4 indicated that resilience did not moderate the relationship between perceived stress and alcohol misuse directly or indirectly because both the interaction effect of perceived stress and resilience on alcohol misuse (*p* = 0.344) and the interaction effect of sleep duration and resilience on alcohol misuse (*p* = 0.917) were not significant. Therefore, the Johnson-Neyman test was not performed for Path 2.

To summarize, the mediation and moderation analysis of Model 4 demonstrated that perceived stress was not associated with alcohol misuse through its relationship with sleep duration. Resilience did not have any effect on the relationship between perceived stress and sleep duration and the relationship between perceived stress and alcohol misuse.

## 4. Discussion

This study examined the complex relationships between perceived stress, dietary behaviors, alcohol misuse, sleep quality and duration, and resilience. Results indicated that sleep quality, but not sleep duration, mediated the relationship between perceived stress and dietary behaviors as well as the relationship between perceived stress and alcohol misuse. Additionally, increased resilience weakened the relationship between perceived stress and dietary behaviors, but resilience did not serve as a buffer to alter the relationship between perceived stress and alcohol misuse. Therefore, improving sleep quality and resilience among higher education students are likely to reduce poor dietary behaviors especially during a high stress situation, like the COVID-19 pandemic. Additionally, improving sleep quality would also appear likely to reduce alcohol misuse among higher education students. However, these possible interventions will need to be empirically tested.

### 4.1. Mediation Effects of Sleep Quality and Sleep Duration on the Relationship between Perceived Stress and Dietary Behaviors

As hypothesized, sleep quality mediated the relationship between perceived stress and dietary behavior. Previous work reported that stress negatively impacts dietary behaviors through reward signal pathways in the brain [47], where high stress motivates consumption of highly palatable foods. While there are many factors that influence the reward system in the brain, sleep plays a major role in its regulation [48,49,50]. Poor sleep quality heightens reward system responses [51], which results in higher consumption of energy and added sugar [52,53] and lower intake of fruits and vegetables [54]. Thus, the role of sleep as a mediator in the relationship of perceived stress and dietary behaviors has a plausible physiological mechanism.

Like poor sleep quality, insufficient sleep also results in poor dietary behaviors [19,20,21,55,56,57,58,59,60]. This may be explained by the fact that, like poor sleep quality, insufficient sleep also amplifies the brain’s reward system [49,50]. However, the present study reported a non-significant mediation effect of sleep duration on the relationship between perceived stress and dietary behaviors. This null finding could be explained by the fact that the average sleep duration of the students (7.5 ± 1.2 h) exceeded the minimum recommended sleep duration of 7 h per day [44]. Previous large cross-sectional studies reported similar results compared to the present study, where students living in different countries, including China [61], the Netherlands [62] and the U.S. [62], generally reported sufficient sleep. Higher education students tend to get enough sleep, on average, when counting both weekday and weekend sleeping hours since students engage in catch-up sleep during the weekends or when schedules allow [63,64,65]. Unlike the present study, previous studies noting a significant relationship between sleep duration and poor dietary behaviors frequently reported insufficient sleep in the study populations [19,20,21,55]. Thus, adequate sleep duration of students in the present study could be the reason why sleep duration did not mediate the relationship between stress and dietary behaviors.

### 4.2. Mediation Effects of Sleep Quality and Sleep Duration on the Relationship between Perceived Stress and Alcohol Misuse

As hypothesized, sleep quality mediated the relationship between perceived stress and alcohol misuse. This finding is consistent with other work which reported that sleep quality mediated the relationship between poor mental health, which included depression, anxiety, and stress, as well as frequent alcohol use [66]. Alcohol consumption among 18 to 25 year-olds is widespread and is commonly used as a strategy to cope with stress [67]. While stress can trigger more frequent alcohol misuse, stress also leads to poor quality sleep [16,18]. Additionally, the influence of sleep on alcohol misuse behaviors has been attributed, in part, to the alteration of neurocognitive function by poor sleep [68]. Poor sleep reduces neural activity in the prefrontal cortex, which carries out higher order executive functions, such as decision making, inhibitory function, and self-monitoring [68,69,70,71]. Thus, sub-optimal executive function may lead to dangerous behaviors, such as excessive drinking, when sleep is compromised [72]. Further, a study of university students revealed that poor sleep predicted drinking to cope with stress [73], which can potentially set up a vicious cycle since frequent alcohol consumption negatively influences sleep quality by disrupting normal sleep cycles [74]. Therefore, sleep quality serves as an intermediate factor that explains why perceived stress and alcohol misuse are connected.

Contrary to our hypothesis, sleep duration did not mediate the relationship between perceived stress and alcohol misuse. Like poor sleep quality, insufficient sleep has been shown to alter the decision-making process, leading to more frequent risky behaviors [68,69,71,75]. The failure to observe sleep duration as a mediator between stress and alcohol misuse in the present study could, again, be attributed to the fact that the student cohort reported sufficient sleep. Unlike the present study, previous work where associations between sleep duration and alcohol misuse were observed frequently reported insufficient sleep among participants [76,77,78]. Taken together, sufficient sleep observed among the present student cohort could explain the non-significant mediation effect of sleep duration on the relationship between perceived stress and alcohol misuse.

Despite students having sufficient sleep on average in the present study, the average sleep quality was poor, which is consistent with other studies [2,79,80,81,82]. The present study occurred during the beginning of the COVID-19 pandemic, when many locations surveyed adopted “shelter in place” orders, and the majority of the students were under quarantine, which could change sleep patterns [83,84,85,86]. As shown in the present study, 44.6% of students reported sleeping more during the COVID-19 pandemic compared to before. Additionally, recent studies noted that university students spent more time in bed during the pandemic compared to before but with poorer sleep quality [84,85,86], which is also consistent with our finding that nearly one-third of students reported worsened sleep quality. Previous work shows that longer sleep duration was related to poorer subjective sleep quality [87]; thus, it is possible that the adequate sleep duration observed in the present student cohort reflects poor sleep quality. Taken together, our findings and the previous evidence suggest poor sleep quality may be an emergent issue to be addressed among higher education students in order to reduce the negative effect of stress on dietary behaviors and the negative effect of stress on alcohol misuse.

### 4.3. Moderation Effects—Resilience

Besides sleep quality, resilience has been noted to play an important role in reducing the negative effect of stress on dietary behaviors [23,24], and this was corroborated in the present study. Higher resilience weakened the relationship between stress and undesirable dietary behaviors, which could be explained by resilience serving as a positive coping strategy for stress [88,89]. Previous work showed that individuals who display greater resilience cope better with adversity; therefore, the negative effects of stressful events, like poor dietary behaviors, are reduced with increased resilience [23,24]. Thus, higher education students are likely to benefit from improved resilience to reduce the negative effect of stress on dietary behaviors.

Contrary to our hypothesis, the present study noted that resilience did not play a role in reducing the negative effect of stress on alcohol misuse. While alcohol misuse can be a consequence of stress [14], resilience did not moderate the relationship between perceived stress and alcohol misuse, which could be attributed to the lower alcohol misuse among the student cohort in the present study during the COVID-19 pandemic compared to previous studies [36,90,91,92,93]. In the present study, most students reported either reduced or no change in alcohol consumption during the COVID-19 pandemic compared to before. Among higher education students, socializing is the most important driving factor for alcohol consumption [94,95]. The COVID-19 pandemic reduced the opportunity for socializing among higher education students, and many students changed living situations, which could further restrict socializing [96]. Thus, these factors likely contributed to lower risk of alcohol misuse among higher education students during the COVID-19 pandemic. In summary, the non-significant moderating effect of resilience on the relationship between perceived stress and alcohol misuse is likely attributed to the lower alcohol misuse among higher education students during the COVID-19 pandemic.

### 4.4. Public Health Messages

Findings from the present study suggest that poor sleep quality is associated with health concerns. Currently, interventions for sleep quality improvement have received little attention by those working with higher education students [97,98]. Poor sleep quality is not only associated with poor dietary behaviors and alcohol misuse, but also associated with poor academic performance [99,100], obesity [101], higher risk of chronic diseases [102], and poorer overall health [103]. One large study of higher education students showed that sleep disturbance had a stronger association with academic success compared to stress, binge drinking, and drug use; however, addressing sleep concerns in the student population is not routinely, or even frequently, done [104]. The present study showed that improving sleep quality may be an effective strategy to improve the health of higher education students and warrants further exploration of how improving sleep quality can improve other health outcomes.

Sleep hygiene education, cognitive behavioral therapy (CBT), relaxation, mindfulness, and hypnotherapy are effective strategies to improve sleep among higher education students [105,106,107]; thus, universities should consider incorporating these strategies to improve sleep quality of these students. One systematic review indicated increased effectiveness of CBT compared to other interventions in improving sleep quality and duration [107], and another systematic review showed that online CBT for insomnia is effective in improving sleep quality and duration among adults with chronic insomnia by identifying and modifying factors that contribute to poor sleep, including stress [108]. Given that students suffered from a decline in sleep quality during the pandemic [84,85,86], an online CBT sleep education program can serve as a feasible way to address a critical health concern of higher education students. As poor sleep quality mediated the relationship between stress and dietary behaviors and the relationship between stress and alcohol misuse, improving sleep quality during a stressful event may prevent health decline of higher education students during this time. Thus, universities should consider initiating and adopting a feasible form of sleep education to improve the health of higher education students.

Higher education students are also likely to benefit from receiving resilience training to improve health. Resilience training is more commonly used among healthcare and medical students rather than general higher education students [109,110]. Limited research conducted among general higher education students indicated that resilience training was just as effective as CBT in improvement of mental health among university students [111], and one interventional study showed that a mindfulness-based intervention to increase resilience significantly decreased stress compared to traditional mental health support offered by university counseling centers [112]. Therefore, resilience training should be considered as a student service in university counseling centers given that it shows promise in reducing stress.

### 4.5. Strengths and Limitations

There are several strengths to this study. First, the large sample size provided adequate power to conduct the mediation and moderation analyses. Second, using the mediation and moderation analyses allowed for the exploration of more complex relationships known to exist between stress, dietary behaviors, alcohol misuse, sleep, and resilience. Third, the study included students from Asia, Europe, and North America and included both undergraduate and graduate students, which increased the generalizability of the results. Finally, the study used validated questionnaires.

There are limitations to the study. First, the cross-sectional nature of the study does not allow for the attribution of causality between the variables examined. Second, weight and height were self-reported because in-person testing was not allowed during the pandemic, and the possibility of mis-reporting cannot be ruled out. Third, the social-restriction rules were different among the locations surveyed during the data collection period. Due to the complexity of social restriction rules among and even within locations, it is difficult to include social restrictions as a covariate; however, the information was collected and reported in Table 2. Fourth, the STC questionnaire, which measures eating frequencies of both healthy and unhealthy foods, does not measure problematic eating behaviors such as uncontrolled or emotional eating. Thus, the dietary behaviors construct presented in this paper only refers to eating frequencies of certain foods. Fifth, questions regarding how the COVID-19 pandemic impacted health behaviors and mental health of higher education students were not validated due to there being no validated questionnaires available when the study was conducted. Sixth, most students in the study self-identified as female undergraduate domestic students, and students from the U.S. and Taiwan were over-represented compared to other participants in this study. The sample population in the study could limit the generalizability of the study results. Seventh, information regarding mental illness and treatment was not collected in the survey due to privacy concerns; therefore, mental illness diagnoses and treatments were not adjusted for in the models examined. Finally, all surveys were administered in English, which restricted the participation of students with low English proficiency.

## 5. Conclusions

The present study identified that sleep quality, but not sleep duration, mediated the relationship between perceived stress and dietary behaviors and the relationship between perceived stress and alcohol misuse. Additionally, increased resilience weakened the relationship between perceived stress and dietary behaviors; the negative effect of high-perceived stress on dietary behaviors disappeared at higher resilience scores. These findings suggest that incorporating sleep quality improvement training and resilience training into university health promotion programs would likely be effective strategies to improve the health of higher education students, especially during stressful events. However, these conclusions need to be further tested with interventional approaches.

## Figures and Tables

**Table 1:** Demographic and biological information.

Sex *n* (%)	Undergraduate vs. Graduate *n* (%)	Domestic vs. International *n* (%)	Age (y) Mean ± SD	BMI (kg/m^2^) Mean ± SD
M = 694 (30.8) F = 1502 (66.7) Other * = 44 (2.0) UD = 14 (0.5) Total = 2254	U = 1802 (79.9) G = 452 (20.1)	D = 1962 (87.0) I = 292 (13.0)	22.5 ± 5.5	24.4 ± 5.6

Note: *n* = number of participants; M = male, F = female, UD = undisclosed; U = undergraduate students, G = graduate students; D = domestic students, I = international students; BMI = body mass index; SD = standard deviation. * Other included transgender, genderqueer, additional gender category or other, and choose not to disclose.

**Table 2:** Social restriction information and sample size by location.

Location	Social Restriction Measures in Place	*n* (% to Total)
China	Mixture of online and return to in-person classes	111 (4.9)
Ireland	Online classes only	192 (8.5)
Malaysia	Online classes only	91 (4.0)
South Korea	Mixture of online and return to in-person classes	89 (3.9)
Taiwan	Mixture of online and return to in-person classes	377 (15.0)
The Netherlands	Online classes only	114 (5.1)
United States	Online classes only	1278 (58.7)

Note: universities surveyed included Hangzhou Normal University in China; Athlone Institute of Technology (AIT), Institute of Technology Sligo (IT Sligo), Letterkenny Institute of Technology (LYIT), Trinity College Dublin (TCD), University of Limerick (UL), Waterford Institute of Technology (WIT), Institute of Technology Tralee (ITT), Dublin City University (DCU), University College Dublin (UCD), Hibernia College, National University of Galway (NUIG), Technological University Dublin (TUD/TU Dublin), Cork Institute of Technology (CIT), Galway-Mayo Institute of Technology (GMIT), University College Cork (UCC), and Griffith College Dublin in Ireland; International Medical University (IMU) in Kuala, Lumpur, Malaysia; Hanyang University, Chungnam National University, Seokyeong University, and University of Seoul in South Korea; University of Taipei in Taiwan; Leiden University College in the Netherlands; Michigan State University, Bowling Green State University, and Indiana University of Pennsylvania in the United States.

**Table 3:** Zero-order correlations between constructs tested and covariates.

Measures ^a^	Mean ± SD	1	2	3	4	5	6	7	8
(1) Dietary behaviors (STC scores)	7.8 ± 2.8	-	0.08	0.20 *	0.18 *	0.02	−0.20 *	−0.04	0.18 *
(2) Alcohol misuse (AUDIT-C scores)	3.1 ± 2.7		-	0.09 *	0.14 *	−0.02	0.002	−0.04	0.12 *
(3) Perceived stress (PSS-10 scores)	20.6 ± 6.8			-	0.43 *	−0.05	−0.50 *	−0.12 *	0.12 *
(4) Sleep quality (PSQI scores)	6.8 ± 3.5				-	−0.33 *	−0.28 *	0.04	0.19 *
(5) Sleep duration (hours)	7.5 ± 1.2					-	0.05	−0.11 *	−0.06
(6) Resilience (BRS scores)	3.2 ± 0.7						-	0.09 *	−0.03
(7) Age (years)	22.5 ± 5.5							-	0.17 *
(8) BMI (kg/m^2^)	24.4 ± 5.6								-

^a^ Numbers in parentheses correspond to column numbers. * Indicates a significant correlation at the *p* < 0.0018 level based on Bonferroni adjustment for multiple comparisons. STC = Starting the Conversation, higher scores indicate poorer dietary behaviors (range 0–16); AUDIT-C = Alcohol Use Disorders Identification Test Alcohol Consumption, higher scores indicate more frequent alcohol misuse (range 0–12); PSS-10 = Perceived Stress Scale-10, higher scores indicate greater stress (range 0–40); PSQI = Pittsburgh Sleep Quality Index, higher scores indicate worse sleep quality (range 0–21); BRS = Brief Resilience Scale, higher scores indicate greater resilience (range 0–5); BMI = body mass index; SD = standard deviation.

**Table 4:** Distribution of health behavior classifications.

	Alcohol Misuse	Stress Levels	Sleep Quality	Sleep Duration
Percentage of students for each classification ^†^	22.8% misuse female 32.1% misuse male 77.2% no misuse female 67.9% no misuse male	15.0% low stress 63.2% moderate stress 21.8% high stress	60.0% poor sleeper 40.0% good sleeper	72.2% met sleep duration 27.8% did not meet sleep duration

^†^ Alcohol misuse was classified as an AUDIT-C score of 4 (of 12) and higher for men, and 3 (of 12) and higher for women. Stress levels were classified using PSS-10 scores, and scores from 0 to 13, 14 to 26, and 27 to 40 indicate low, moderate, and high levels of stress, respectively. Poor sleepers were classified using the PSQI scores being ≥5 (of 21). Students who reported sleeping at least 7 h per day were classified as meeting sleep duration guidelines.

**Table 5:** Distribution of health behaviors, perceived stress, and resilience changes during the COVID-19 pandemic.

	Diet	Sleep Quality		Alcohol Use *	Sleep Duration	Perceived Stress	Resilience
Worse	35.2%	32.0%	More	16.8%	44.6%	60.2%	19.5%
Better	26.6%	15.5%	Less	21.6%	17.1%	13.0%	29.5%
No change	38.2%	52.3%	No change	39.7%	38.2%	26.8%	51.0%
Net effect	8.6% worse	16.5% worse		4.8% better	27.5% better	47.2% worse	10.0% worse

* Alcohol consumption *n* = 1761; 21.9% (*n* = 386) students reported they do not consume alcohol.

**Table 6:** Model 1 mediation analysis.

**Variables**	**B**	**SE**	**t**	***p* Value**
Perceived stress → sleep quality	0.35	0.04	8.42	<0.001
Sleep quality → dietary behaviors	0.01	0.08	0.11	0.916
Perceived stress → dietary behaviors	0.12	0.04	3.12	0.002
**Bootstrap**	**Effect**	**SE**	**LL 95%CI**	**UL 95% CI**
Sleep quality	0.02	0.004	0.008	0.023

**Table 7:** Model 1 moderation analysis.

Variables				
**Moderation Path 1**	**B**	**SE**	**t**	***p*** **Value**
Resilience → sleep quality	−0.65	0.29	−2.21	0.03
Perceived stress × resilience → sleep quality	−0.05	0.01	−4.35	<0.001
**Moderation Path 2**	**B**	**SE**	**t**	***p*** **Value**
Perceived stress × resilience → dietary behaviors	−0.03	0.01	−2.27	0.023
Sleep quality × resilience → dietary behaviors	0.02	0.02	1.01	0.313
**Conditional Indirect Effect of Resilience in Moderation Path 1**	**Resilience Scores**	**Effect (SE)**	**LL 95% CI**	**UL 95% CI**
	2.44	0.22 (0.02)	0.19	0.25
	3.21	0.18 (0.01)	0.16	0.21
	3.97	0.14 (0.01)	0.12	0.17
**Conditional Indirect Effect of Resilience** **Johnson-Neyman Test**	**Resilience Scores**	**Indirect Effect (SE)**	**LL 95% CI**	**UL 95% CI**
	1.00	0.30 (0.03)	0.24	0.36
	1.80	0.26 (0.02)	0.21	0.30
	2.60	0.21 (0.02)	0.19	0.24
	3.40	0.17 (0.01)	0.15	0.20
	4.20	0.13 (0.02)	0.10	0.16
	4.40	0.12 (0.02)	0.09	0.16
	4.60	0.11 (0.02)	0.07	0.15
	4.80	0.10 (0.02)	0.06	0.14
	5.00	0.09 (0.02)	0.05	0.14
**Conditional Direct Effect of Resilience** **in Moderation Path 2**	**Resilience Scores**	**Effect (SE)**	**LL 95% CI**	**UL 95% CI**
	2.44	0.06 (0.01)	0.03	0.09
	3.21	0.04 (0.01)	0.02	0.06
	3.97	0.02 (0.01)	−0.004	0.05
**Conditional Direct Effect of Resilience** **Johnson-Neyman Test**	**Resilience Scores**	**Direct Effect (SE)**	**LL 95% CI**	**UL 95% CI**
	1.00	0.10 (0.03)	0.04	0.15
	1.80	0.08 (0.02)	0.04	0.12
	2.60	0.06 (0.01)	0.03	0.08
	3.40	0.04 (0.01)	0.01	0.06
	3.87 *	0.02 (0.01)	0.00	0.05
	4.40	0.01 (0.02)	−0.02	0.04
	4.60	0.01(0.02)	−0.03	0.04
	4.80	0.001 (0.02)	−0.04	0.04
	5.00	−0.005 (0.02)	−0.05	0.04

* *p* = 0.05.

**Table 8:** Model 2 mediation analysis.

**Variables**	**B**	**SE**	**t**	***p*** **Value**
Perceived stress → sleep duration	−0.04	0.02	−2.79	0.005
Sleep duration → dietary behaviors	0.06	0.18	0.35	0.726
Perceived stress → dietary behaviors	0.12	0.03	3.59	<0.001
**Bootstrap**	**Effect**	**SE**	**LL 95% CI**	**UL 95% CI**
Sleep duration	−0.002	0.001	−0.004	0.0002

**Table 9:** Model 2 moderation analysis.

Variables				
**Moderation Path 1**	**B**	**SE**	**t**	***p*** **Value**
Resilience → sleep duration	−0.21	0.11	−1.89	0.06
Perceived stress × resilience → sleep duration	0.01	0.005	1.72	0.09
**Moderation Path 2**	**B**	**SE**	**t**	***p*** **Value**
Resilience → dietary behaviors	0.01	0.50	0.03	0.978
Perceived stress × resilience → dietary behaviors	−0.02	0.01	−2.41	0.016
Sleep duration × resilience → dietary behaviors	0.01	0.06	0.19	0.851
**Conditional Indirect Effect of Resilience** **in Moderation Path 1**	**Resilience Scores**	**Effect (SE)**	**LL 95% CI**	**UL 95% CI**
	2.44	−0.03 (0.01)	−0.04	−0.01
	3.21	−0.02 (0.01)	−0.03	−0.01
	3.97	−0.01 (0.01)	−0.02	−0.002
**Conditional Direct Effect of Resilience** **in Moderation Path 2**	**Resilience Scores**	**Effect (SE)**	**LL 95% CI**	**UL 95% CI**
	2.44	0.07 (0.01)	0.04	0.09
	3.21	0.05 (0.01)	0.03	0.07
	3.97	0.03 (0.01)	0.01	0.05
**Conditional Direct Effect of Resilience** **Johnson-Neyman test**	**Resilience Scores**	**Direct Effect (SE)**	**LL 95% CI**	**UL 95% CI**
	1.00	0.10 (0.03)	0.05	0.15
	1.80	0.08 (0.02)	0.05	0.12
	2.60	0.06 (0.01)	0.04	0.09
	3.40	0.04 (0.01)	0.02	0.06
	4.16 *	0.03 (0.01)	0.00	0.05
	4.40	0.02 (0.01)	−0.01	0.05
	4.60	0.01(0.02)	−0.02	0.05
	4.80	0.01 (0.02)	−0.03	0.04
	5.00	0.005 (0.02)	−0.03	0.04

* *p* = 0.05.

**Table 10:** Model 3 mediation analysis.

**Variables**	**B**	**SE**	**t**	***p*** **Value**
Perceived stress → sleep quality	0.39	0.05	8.44	<0.001
Sleep quality → alcohol misuse	0.14	0.08	1.60	0.109
Perceived stress → alcohol misuse	−0.05	0.04	−1.04	0.299
**Bootstrap**	**Effect**	**SE**	**LL 95% CI**	**UL 95% CI**
Sleep quality	0.02	0.004	0.009	0.025

**Table 11:** Model 3 moderation analysis.

Variables				
**Moderation Path 1**	**B**	**SE**	**t**	***p*** **Value**
Resilience → sleep quality	−0.93	0.32	−2.91	0.004
Perceived stress × resilience → sleep quality	−0.06	0.01	−4.55	<0.001
**Moderation Path 2**	**B**	**SE**	**t**	***p*** **Value**
Resilience → alcohol misuse	−0.08	0.28	−0.29	0.771
Perceived stress × resilience → alcohol misuse	0.02	0.01	1.59	0.112
Sleep quality × resilience → alcohol misuse	−0.01	0.03	−0.56	0.573
**Conditional Indirect Effect of Resilience** **in Moderation Path 1**	**Resilience Scores**	**Effect (SE)**	**LL 95% CI**	**UL 95% CI**
	2.49	0.23 (0.02)	0.20	0.27
	3.18	0.19 (0.01)	0.16	0.21
	3.88	0.14 (0.02)	0.12	0.18
**Conditional Indirect Effect of Resilience** **Johnson-Neyman Test**	**Resilience Scores**	**Indirect Effect (SE)**	**LL 95% CI**	**UL 95% CI**
	1.00	0.33(0.03)	0.26	0.39
	1.80	0.28 (0.02)	0.23	0.32
	2.60	0.23 (0.02)	0.20	0.26
	3.40	0.18 (0.01)	0.15	0.20
	4.20	0.13 (0.02)	0.09	0.16
	4.40	0.12 (0.02)	0.07	0.15
	4.60	0.10 (0.02)	0.06	0.15
	4.80	0.09 (0.03)	0.04	0.14
	5.00	0.08 (0.03)	0.02	0.13
**Conditional Direct Effect of resilience** **in moderation path 2**	**Resilience Scores**	**Effect (SE)**	**LL 95% CI**	**UL 95% CI**
	2.49	0.01 (0.02)	−0.02	0.04
	3.18	0.02 (0.01)	−0.003	0.04
	3.88	0.03 (0.02)	0.01	0.06

**Table 12:** Model 4 mediation analysis.

**Variables**	**B**	**SE**	**t**	***p*** **Value**
Perceived stress → sleep duration	−0.02	0.02	−1.02	0.310
Sleep duration → alcohol misuse	−0.06	0.21	−0.27	0.789
Perceived stress → alcohol misuse	0.001	0.04	0.03	0.975
**Bootstrap**	**Effect**	**SE**	**LL 95% CI**	**UL 95% CI**
Sleep duration	0.0002	0.0004	−0.0006	0.0013

**Table 13:** Model 4 moderation analysis.

Variables				
**Moderation Path 1**	**B**	**SE**	**t**	***p*** **Value**
Resilience → sleep duration	0.01	0.12	−1.02	0.310
Perceived stress × resilience → sleep duration	0.004	0.01	0.79	0.431
**Moderation Path 2**	**B**	**SE**	**t**	***p*** **Value**
Resilience → alcohol misuse	−0.07	0.58	−0.13	0.900
Perceived stress × resilience → alcohol misuse	0.01	0.01	0.95	0.344
Sleep duration × resilience → alcohol misuse	0.01	0.07	0.10	0.917
**Conditional Direct Effect of Resilience** **in Moderation Path 2**	**Resilience Scores**	**Effect (SE)**	**LL 95% CI**	**UL 95% CI**
	2.49	0.03 (0.02)	0.001	0.06
	3.18	0.04 (0.01)	0.02	0.06
	3.88	0.04 (0.01)	0.03	0.07

## Data Availability

The data presented in this study are available on request from the corresponding author. The data are not publicly available due to ongoing analyses.
